# Anthracycline therapy induces an early decline of cardiac contractility in low-risk patients with breast cancer

**DOI:** 10.1186/s40959-024-00244-y

**Published:** 2024-07-16

**Authors:** Fabian Voß, Fabian Nienhaus, Saskia Pietrucha, Eugen Ruckhäberle, Tanja Fehm, Tobias Melz, Mareike Cramer, Sebastian M. Haberkorn, Ulrich Flögel, Ralf Westenfeld, Daniel Scheiber, Christian Jung, Malte Kelm, Amin Polzin, Florian Bönner

**Affiliations:** 1 Division of Cardiology, Pulmonology, and Vascular Medicine, Medical Faculty, University Hospital Düsseldorf, Moorenstr. 5, 40225 Düsseldorf, Germany; 2 Division of Gynecology, Medical Faculty, University Hospital Düsseldorf, Düsseldorf, Germany; 3 Medical Faculty, CARID, Cardiovascular Research Institute Düsseldorf, Heinrich-Heine-University, Moorenstrasse 5, 40225 Düsseldorf, Germany; 4 Department of Molecular Cardiology and Cardiovascular Research Institute Düsseldorf, Medical Faculty, University Hospital Düsseldorf, Düsseldorf, Germany

**Keywords:** Breast cancer, Anthracyclines, Cardiotoxicity, Myocardial damage, CMR

## Abstract

**Aims:**

Cancer therapy-related cardiac dysfunction (CTRCD) is a dreaded complication of anthracycline therapy. CTRCD most frequently appears in patients with cardiovascular risk factors (CVR) or known cardiovascular disease. However, limited data exist on incidence and course of anthracycline-induced CTRCD in patients without preexisting risk factors.

We therefore aimed to longitudinally investigate a cohort of young women on anthracycline treatment due to breast cancer without cardiovascular risk factors or known cardiovascular disease (NCT03940625).

**Methods and results:**

We enrolled 59 women with primary breast cancer and scheduled anthracycline-based therapy, but without CVR or preexisting cardiovascular disease. We conducted a longitudinal assessment before, immediately and 12 months after cancer therapy with general laboratory, electrocardiograms, echocardiography and cardiovascular magnetic resonance (CMR), including myocardial relaxometry with T1, T2 and extracellular volume mapping.

Every single patient experienced a drop in CMR-measured left ventricular ejection fraction (LVEF) of 6 ± 3% immediately after cancer therapy. According to the novel definition 32 patients (54.2%) developed CTRCD after 12 months defined by reduction in LVEF, global longitudinal strain (GLS) and/or biomarkers elevation, two of them were symptomatic. Global myocardial T2 relaxation times as well as myocardial mass increased coincidently with a decline in wall-thickening. While T2 values and myocardial mass normalized after 12 months, LVEF and GLS remained impaired.

**Conclusion:**

In every single patient anthracyclines induce a decline of myocardial contractility, even among patients without pre-existing risk factors for CTRCD. Our data suggest to thoroughly evaluate whether this may lead to an increased risk of future cardiovascular events.

**Graphical Abstract:**

Reduced myocardial contractility in low-risk patients receiving anthracycline-based cancer therapy.

This study included 59 otherwise healthy women with primary breast cancer undergoing anthracycline-based chemotherapy. CMR was performed at baseline, directly and 12 months after cancer therapy. A decline in left ventricular function was observed in every single patient accompanied by transient edema. More than 50% were diagnosed with cancer therapy related cardiovascular dysfunction.

LVEF: left ventricular function, CTRCD: cancer therapy related cardiovascular dysfunction, GLS = Global longitudinal strain, hs-TnT = high sensitive Troponin T, NT-pro BNP = NT-pro brain natriuretic peptide

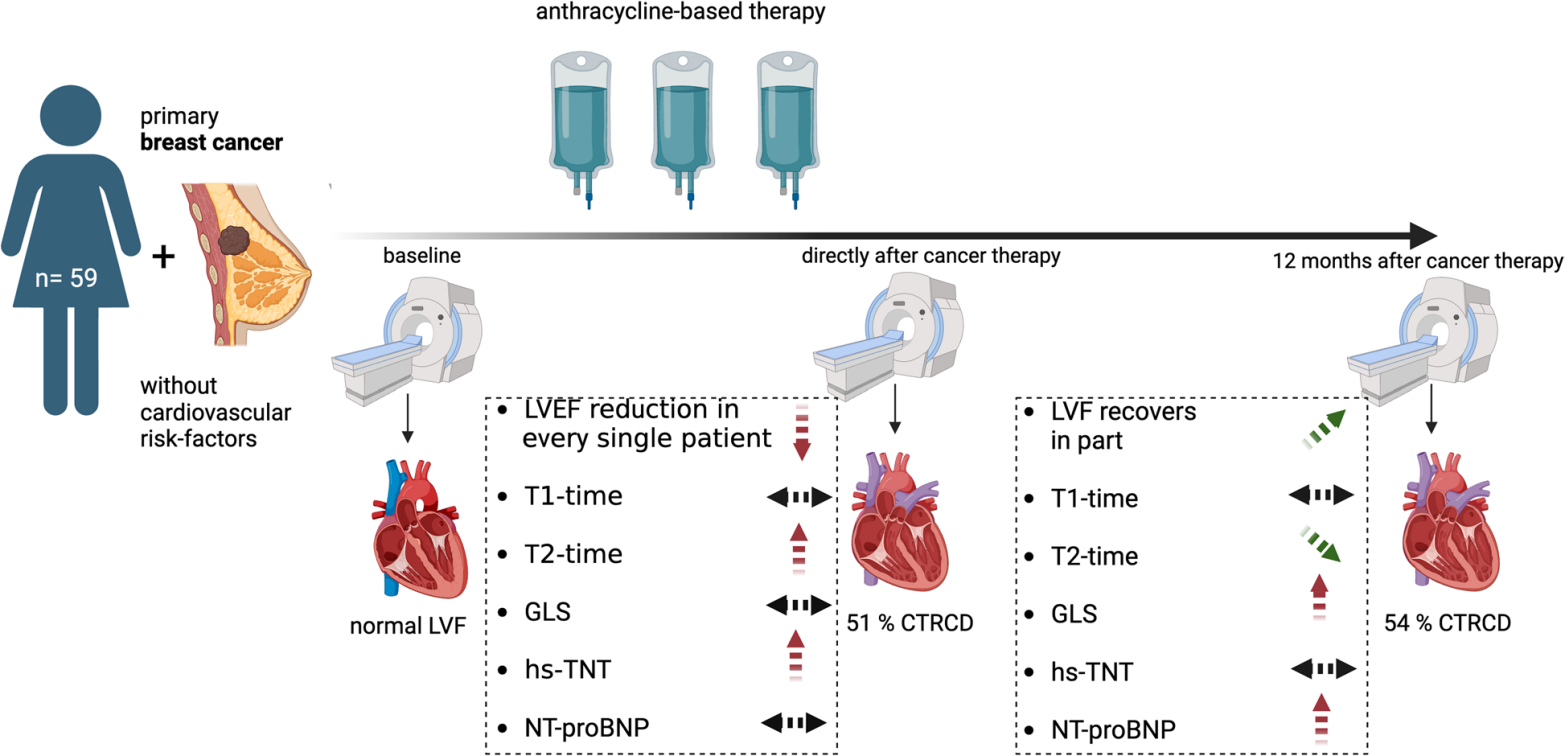

**Supplementary Information:**

The online version contains supplementary material available at 10.1186/s40959-024-00244-y.

## Lay summery

Anthracyclines are a corner stone of the conventional chemotherapies used to treat breast cancer. Cardiotoxicity is a known side effect of this highly effective therapy which has contributed to the high numbers of patients who can be healed from breast cancer nowadays. Consequently, many patients suffer from cardiovascular diseases after surviving their cancer diagnosis and treatment. It is therefore of utmost importance to prevent patients from these side effects whenever possible.

Earlier studies, with only few exceptions, did focus on patients with pre-known risk factor for cardiotoxicity such as diabetes mellitus or arterial hypertension. Our study focused on otherwise healthy women with first diagnosed breast cancer, using cardiac magnetic resonance. Over a follow-up period of one year more than every second patient developed, an in part reversible, cardiotoxicity according to the current guidelines.

Our results therefore suggest that close monitoring, even among healthy subjects, is of utmost importance and further studies are needed to address long term implications of these results.

## Introduction

Breast cancer is the most common female cancer worldwide with an estimated incidence of 2.3 million cases per year in the United States [1, 2]. Besides surgical removal and targeted therapies in selected tumors, anthracycline based chemotherapy remains a corner stone of breast cancer therapy. Those continuously improving therapies, accompanied by the introduction of screening programs, have led to high rates of patients who can be cured from their life-threatening disease. Nowadays, there is an overall 5-year survival rate of more than 90% in highly developed countries [3]. Since survival rates are increasing, the control of the toxic side effects becomes crucial to further improve patient's life expectancy and quality of life.

Anthracyclines are known to induce cancer therapy-related cardiac dysfunction (CTRCD), which leads to heart failure hospitalization and death in these patients [4]. The molecular background behind CTRCD due to anthracyclines is not fully understood but binding of topoisomerase-2ß leading to the inhibition of mitochondrial biogenesis, the activation of cell death pathways in cardiomyocytes and an increased production of reactive oxygen species leading to lipid peroxidation and DNA damages, have been identified as the most important mechanisms [5–7].

Different risk assessments were established to identify individuals with the highest risk of CTRCD [8]. Older patients and those with cardiovascular risk factors are most prone to develop CTRCD after anthracycline therapy. These individuals should be monitored carefully during their cancer therapy. If CTRCD has developed, specific therapies and in severe cases even interruption of the current cancer therapy must be evaluated according to the recently published guidelines of the European society of cardiology [9].

Although patients at the highest risk for CTRCD have been investigated intensively, little is known about cardiotoxic effects in young breast cancer patients with a low risk for developing CTRCD. Approximately 18% of women are under 50 years at the timepoint of initial diagnosis [2]. In these young patients cardiotoxic effects may impact patient's life for decades.

Therefore, in this study we thought to investigate CTRCD in young patients with breast cancer and anthracycline therapy. We hypothesized that CTRCD, according to the new definitions, is detectable even among these women classified as low risk patients and is reversable after one year after cancer therapy.

## Methods

### Study design and participants

The multi-center study was designed as longitudinal prospective observational cohort study recruiting from 01/2017–01/2020. The participants were recruited at the University Hospital Duesseldorf and at Marienhospital Duesseldorf, Germany. Inclusion criteria were primary breast cancer and planned anthracycline-including therapy. Prespecified exclusion criteria were any contraindications to perform cardiac magnetic resonance (CMR) (claustrophobia, implants without CMR certification, inability to follow instructions during CMR), previous anthracycline therapies for other malignancies, major cardiovascular risk factors (hypertension, hypercholesterolemia, diabetes mellitus), known cardiovascular diseases and a missing written consent. All procedures were performed in accordance with the declaration of Helsinki and the International Conference on Harmonization of Good Clinical Practice. All patients provided written informed consent. The study protocol was approved by the local ethics committee of the University Hospital Düsseldorf (5018R). ClinicalTrials identifier: NCT03940625.

All 59 participants underwent repetitive blood sampling, electrocardiogram, echocardiography and CMR at baseline, immediately (within 3 days) after the last cycle and 12 months after anthracycline therapy (Graphical Abstract).

### CMR data acquisition

CMR was conducted with a 1.5 Tesla scanner (Achieva, Philips, Best, The Netherlands) with a 32-channel phased array coil. Established protocols were used as described before [10, 11] and investigators were blinded concerning cancer therapy and CMR timepoint. In short, the protocol consisted of scout and reference scans followed by cine-imaging in continuous short axis slices covering the whole ventricle, T1 and T2-mapping and ECV estimation.

The following parameters were determined based on the sequences of CMR: left ventricular ejection fraction (LVEF), indexed enddiastolic (EDVi), endsystolic (ESVi), stroke volume, left atrial volume (LAVi), right atrial volume (RAVi), myocardial mass (LVM), cardiac index, resting cardiac power index and myocardial relaxometry with T1 and T2 mapping. Locally established normal ranges are 930–1050 ms for T1- and 55–60 ms (aged < 60 years), respectively 59–65 ms (aged > 60 years) for T2-times. Furthermore, global and local contractility were analyzed using the wall thickening, wall motion and feature tracking derived deformation analysis. In detail global longitudinal (GLS), circumferential (GCS) and radial (GRS) strain analyses were performed. For analysis cvi42© from Circle Cardiovascular Imaging (Calgary, Alberta, Canada) with automated border detection was used. Myocardial T2 relaxation times were measured and analyzed according to previous protocols [11]. Body surface area was calculated using the Dubois method. CMR estimated pulmonary capillary wedge pressure (PCWP) was calculated as described by Garg et al. [12] as follows: Calculated PCWP = 6.1352 + (0.07204 ∗ LAV) + (0.02256 ∗ LVM).

### Echocardiographic data acquisition

Echocardiographic measurements (biplane LVEF-estimation by Simpsons’-method, ESV, EDV, E- and A-wave velocities, e’ septal and lateral, GLS) were performed according to the current recommendations [13] and investigators were blinded to the respective CMR results and timepoints of treatment. GLS was measured using 3-standard views (apical 2-, 3- and 4 chamber views). Examinations were performed using commercially available echocardiography devices (Vivid E95, GE, General Electric Company, Boston, USA) and analyses were performed live on the device. Since the examinations were performed during the daily clinical routine, sonographers may have changed between patients and timepoints reflecting clinical routine imaging and analysis.

### Blood sampling and analysis

Blood was drawn from participants at each timepoint and analyzed for high sensitivity troponin-t (TnT) and NT-proBNP using the commercially available electrochemiluminescence immunoassays with cobas® 8000 module 801 (Roche Holding AG, Basel, Switzerland). Normal values, as determined by the manufacturer, were < 14 ng/l for TnT and < 125 pg/ml for Nt-proBNP. Limit of detection was 5 pg/ml for Nt-proBNP and 5 ng/l for TNT, whereas the limits of blank were 8 pg/ml and 3 ng/l, respectively.

### Statistical analysis

Shapiro–Wilk tests were used to confirm normal distributions among the analyzed data. Data are shown as mean ± standard deviation (SD) or as median with 25 and 75% percentile in cases of non-normal distribution. For categorical variables relative and absolute frequencies are displayed. Data was analyzed using a one-way ANOVA followed by a Tukey’s analysis to correct for multiple comparisons. In cases of missing data linear mixed models were used. For nonparametric distributions Friedman test was used followed by Dunn's post-hoc test to compare individual time points. Wilcoxon test was used to compare pairs of non-parametric variables. All tests were performed using GraphPad Prism 9.0. for mac OS. Sample size has been chosen slightly higher than calculated using a power-calculation performed with G*Power3 for mac OS to overcome dropouts. With an effect-size defined as a mean difference of 2% in LVEF, an estimated SD of 4,1%, as derived from the placebo arm of an earlier study [14] and an alpha-error of 0.05 the analyses revealed a sample size of at least 47 patients to detect changes in repeated measures.

## Results

### Study population, cancer therapy and risk stratification

**Fig. 1:**
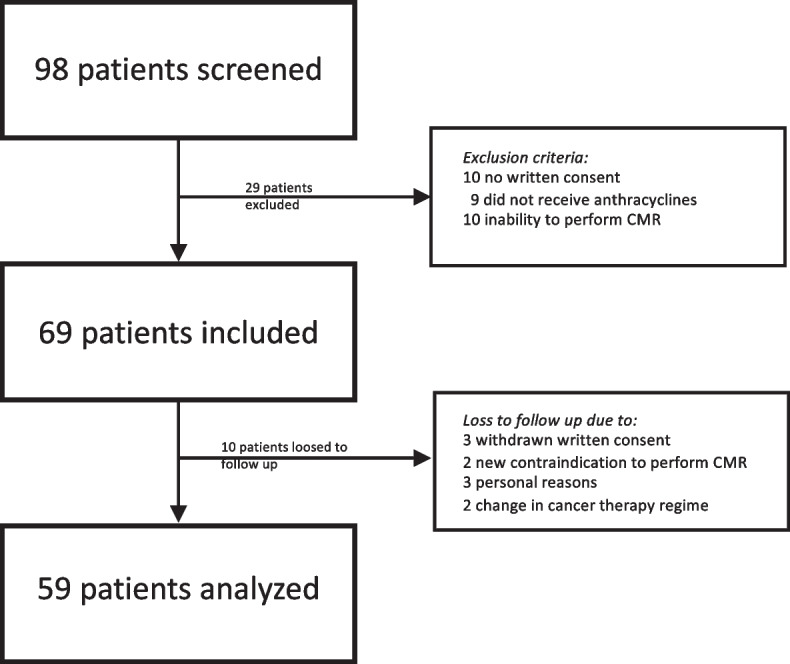
Screening, Inclusion and Follow-up of patients. 98 patients were screened and main reasons for exclusion were missing written consent (*n* = 10), inability to perform CMR (10) and cancer therapy other than anthracyclines (9) whereas reasons for a loss to follow up were a withdrawn written consent (3), new contraindications to perform CMR (2), patient’s personal reasons (3) and a change in cancer therapy (3). Patients were analyzed (59) when at least baseline and one following CMR were performed

After screening of 98 patients, 59 were finally included and analyzed (Fig. 1, baseline CMR *n* = 59, after cancer therapy *n* = 54, after 12 months *n* = 56). Patients had a mean age of 45.47 ± 9.47 years and a body mass index of 24.6 ± 3.8 kg/m^2^ (Table 1). Patients were free of major cardiovascular risk factors including arterial hypertension, hyperlipidemia, diabetes mellitus and prior cardiac diseases (Table 1). A family history of cardiovascular disease existed in a single patient (2%) and ten patients (17%) had a history of smoking but had stopped at the timepoint of cancer diagnosis. No patient had previous cardiotoxic cancer treatments (Table 1). According to the HFA-ICOS risk assessment, as recommended by the ESC guidelines, all patients were classified as low risk for developing CTRCD.

**Table 1:** Baseline characteristics of the study cohort

	Mean ± standard deviation or n (%)
Demographics and cancer-related therapy	
Age, yrs	45,5 ± 9.47
BMI, kg/m^2	24.6 ± 3.8
Breast cancer side	
Left	26 (44)
Right	18 (31)
Cancer therapy	
Cumulative anthracycline dose, epirupicin, mg/m^2	352 ± 13
Paclitaxel dose, mg/m^2	964.4 ± 126.76
Additional therapies	
Carboplatin	11 (19)
Trastuzumab/ Pertuzumab	8 (13)
Radiotherapy	37 (63)
Radiation dose, Gy	52.4 ± 9
Left-sided Radation	24 (41)
Cardiovascular risk factor	
Prior Cardiovascular Disease	0 (0)
Hypertension	0 (0)
Hyperlipidemia	0 (0)
Current Smoking/ Ex-Smoker	0 (0)/10 (17)
Diabetes	0 (0)
Family history of CV disease	1 (2)
Cardiac medications	0 (0)
Previous cardiotoxic cancer treatment	0 (0)
Systolic blood pressure, mm Hg	118 ± 15
Diastolic blood pressure, mm Hg	82 ± 8
Heart rate, beats/min	75 ± 12
Left ventricular ejection fraction, %	63.65 ± 3.2
NT-proBNP level in plasma, pg/m	64 [40.8; 93.5]
Hs-TnT level in plasma,ng/mL	4 [4;5]

Values are presented as mean ± standard deviation or n (%); *BMI* Body mass index

Breast cancer was left sided in 44% of the patients and cumulative epirubicin dose was 352 ± 13 mg/m^2^ (doxorubicin equivalent: 281.6 ± 2.4 mg/m^2^). All patients received a standard therapy regime consisting of epirubicin and cyclophosphamide every three weeks. In 68% paclitaxel was added to the standard therapy. Carboplatin has been added for patients with triple-negative tumors (19%). Patients with a HER2 positive tumor, received monoclonal antibodies (Trastuzumab/Pertuzumab) in addition to the standard therapy (13%) (suppl. Figure 1). In total 63% of the patients received radiotherapy with a radiation dose of 52,4 ± 9 Gy (Table 1).

### Assessment of myocardial function

**Fig. 2:**
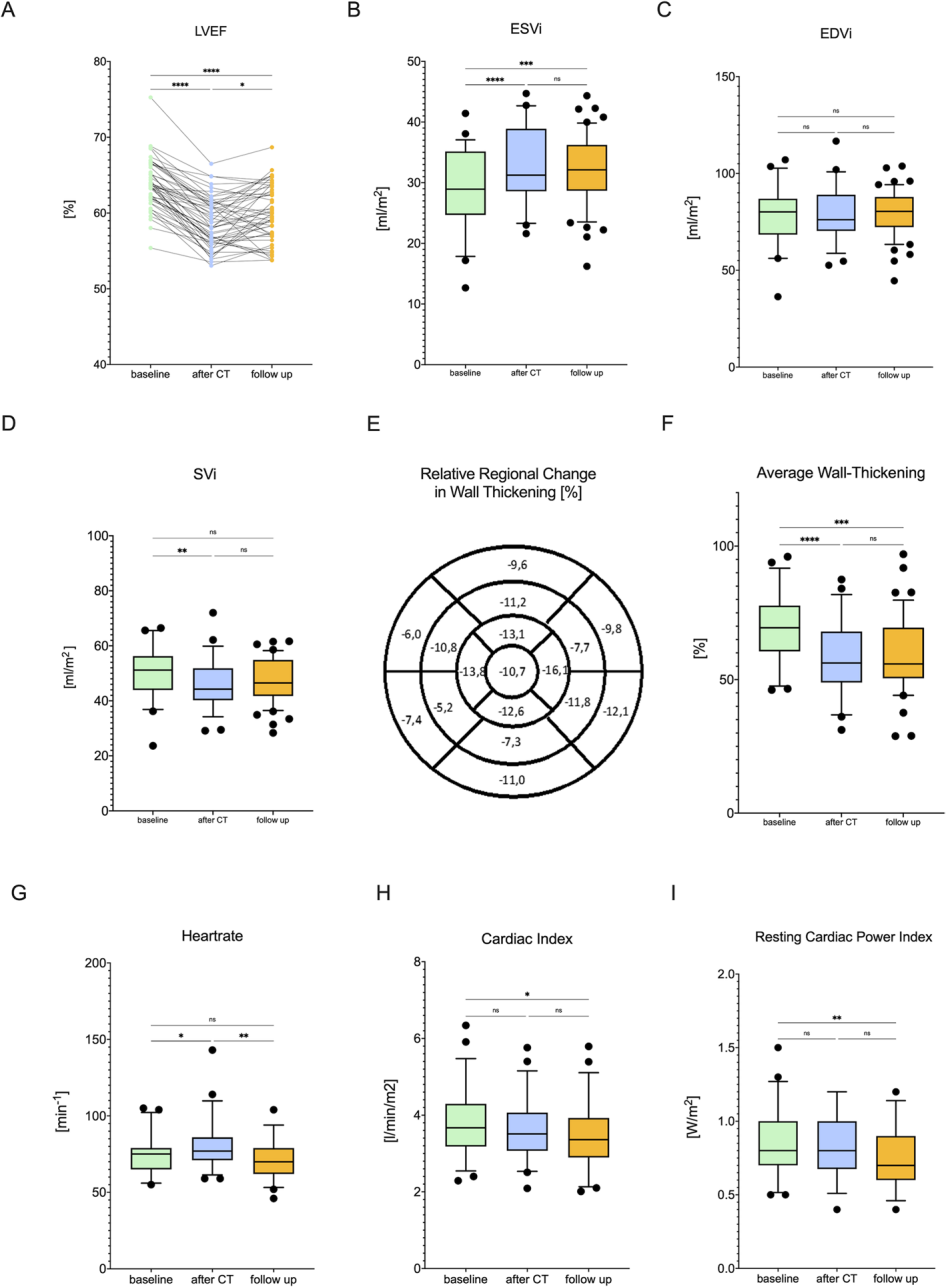
Anthracycline therapy leads to a decline in global systolic function in every single patient with incomplete recovery after 12 months. Shown are measurements of LV systolic function on individual basis (**A**) and LV volumes derived from CMR. Values of ESV (**B**), EDV (**C**) and stroke volume (**D**) were indexed to the patient’s body surface area. All myocardial segments were affected as shown by global (left) and segmentwise (right) wall thickening analysis derived from CMR (12 slices short axis cine view). The segmentwise analysis is presented as difference between baseline and immediately after cancer therapy (**E**) and reduction in average wall-thickening maintained 12 months after cancer therapy (**F**). Heartrate increased directly after cancer therapy but normalized afterwards (**G**) whereas cardiac index (**H**) and resting cardiac power index (**I**) were reduced after 12 months. Data are shown as mean with 95% confidence-interval, statistics are performed by ANOVA for repeated measurements followed by Tukeys post-hoc analyses or linear mixed models in cases of missing data, respectively by Friedmanns test followed by Dunn’s post-hoc analyses in cases of non-normal distribution, *n* = 59. CMR = cardiac magnetic resonance, CI: cardiac index, CO: cardiac output, CPO: cardiac power output, CPI: resting cardiac power index, EDV: end-diastolic volume, ESV: end-systolic volume, LV: left ventricular, SV: stroke volume, *: *p* < 0.05, **: *p* < 0.01, ***: *p* < 0.001, ****: *p* < 0.0001. Figure created with GrahPad Prism 9 for macOS

**Fig. 3:**
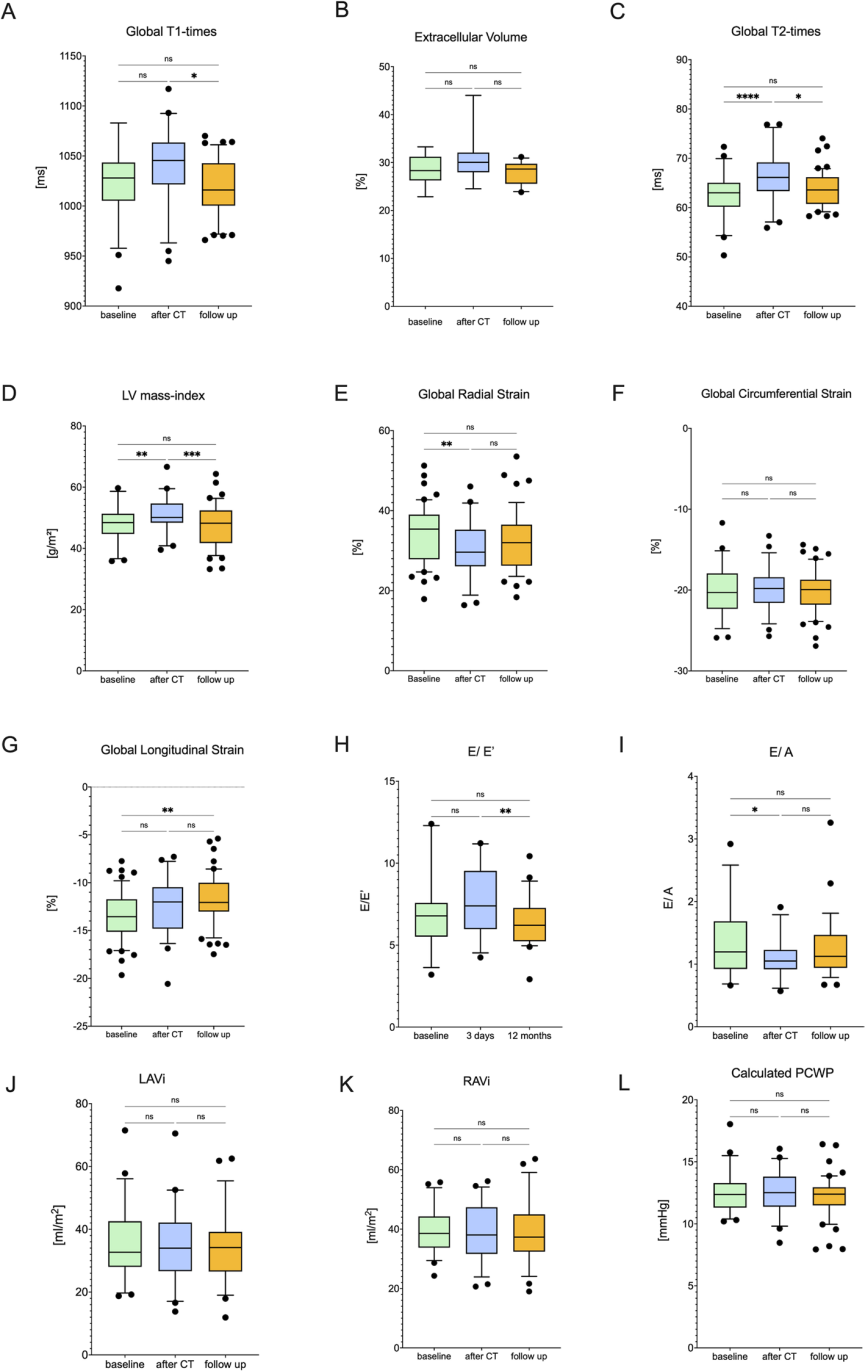
Transient edema leads to increased left ventricular mass and is accompanied by reduction in global longitudinal strain but not by diastolic dysfunction. Global T1-times (**A**) and extracellular volume (**B**) remained stable, whereas T2-times (**C**) showed a transient elevation after cancer therapy followed by an increased indexed LV-mass (**D**). GRS (**E**) showed a transient reduction, whereas GCS (**F**) did not change over time. GLS remained impaired after 12 months (**G**). Echocardiographic measured E/ E’ remained stable (**H**) but E/ A ratio (**I**) showed a transient decrease. Nevertheless, diastolic dysfunction assessed by CMR did not show any changes (LAVi (**J**), RAVi (**K**) and calculated PCWP (**L**)). Data are shown as means with 95% confidence-interval, statistics are performed by ANOVA for repeated measurements followed by Tukeys post-hoc analyses respectively by Friedmanns test followed by Dunn’s post-hoc analyses in cases of non-normal distribution, *n* = 59. Figure created with GrahPad Prism 9 for macOS. CMR = cardiac magnetic resonance, GCS = Global circumferential strain, GLS = Global longitudinal strain, GRS = Global radial strain, LA = Left atrium, LAVi: left atrial volume index, RAVi: right atrial volume index, PCWP: pulmonary capillary wedge pressure *: *p* < 0.05, **: *p* < 0.01, ***: *p* < 0.001, ****: *p* < 0.0001

Myocardial function was primarily assessed by CMR. CMR revealed a drop in every single patient’s LVEF with a mean decline of 6 ± 3% immediately after cancer therapy (Fig. 2A, Table 2). After 12 months of follow up only incomplete recovery has been observed (Fig. 2A). LVEF-reduction was caused by an increase in ESVi whereas EDVi remained stable (Fig. 2B + C). Stroke volume index was reduced after cancer therapy but recovered afterwards (Fig. 2D). An initially increased heart rate normalized after 12 months (Fig. 2G), cardiac index and resting cardiac power index were lower directly and 12 months after cancer therapy (Fig. 2H + I). Systolic wall thickening was consistently reduced in every segment of the left ventricle without regional preference (Fig. 2E, F). Thus, functional impairment of the myocardium appeared not locally, but globally. On strain analyses using CMR feature tracking GRS was reduced initially but recovered in the course, in contrast GCS has not been affected by cancer therapy in our analysis (Fig. 3E + F). Mean GLS did not differ between baseline and directly after cancer therapy but was altered after 12 months (Fig. 3G, absolute difference: 1.78%; relative change:13.15%).

**Table 2:** Volumetric and functional assessment of the left ventricle

Variable	before therapy *n* = 59	after CT *n* = 54		follow up *n* = 56	
			*P* value		*P* value
LVEF, %	63.7 ± 3.2	58.4 ± 3.5	< 0.001	59.9 ± 3.7	< 0.001
EDV/BSA, ml/ m^2^	78.7 ± 13.8	78.3 ± 12.1	0.95	78.3 ± 14.89	0.96
ESV/BSA, ml/ m^2^	28.6 ± 6	33.2 ± 6.6	< 0.001	31.5 ± 7	< 0.001
SV/BSA, ml/ m^2^	49.8 ± 8.7	46.3 ± 8	0.007	47.9 ± 9.7	0.29
Cardiac Index, l/min/m^2^	3.7 ± 0.8	3.6 ± 0.8	0.57	3.4 ± 0.9	0.03
Heart Rate, bpm	75 ± 12	80 ± 15	0.035	72 ± 12	0.64
T1-values, ms	1025 ± 4.8	1041 ± 5.2	0.07	1019 ± 4.3	0.02
T2-values, ms	63 ± 0.6	66.2 ± 0.7	< 0.001	63.7 ± 0.5	0.01
ECV, %	28 ± 0.76	31 ± 1.09	0.09	28 ± 0.59	0.06
LV-Mass Index, g/m^2^	48 ± 0.8	51 ± 0.8	0.004	47 ± 1.1	< 0.001

*BSA* Body surface area, *EDV* End-diastolic volume, *ESV *End-systolic volume, *SV* Stroke volume, *EF* Ejection fraction, *CT* Chemo- and/or targeted therapy, *ECV* Extracellular volume, *LV* Left ventricular

Compared to CMR measurements, echocardiographic-measured LVEF did not change significantly after cancer therapy (baseline: 63.1 ± 0.81, after cancer therapy: 61.8 ± 0.98, after 12 months: 61.2 ± 0.73%, *n* = 43 *p* = 0.17). The same applied for EDV (81.7 ± 2.87 vs. 89.9 ± 3,19 vs. 86.3 ± 4,38 ml, *p* = 0.21) and ESV (31.3 ± 1.58 vs. 34 ± 1.71 vs. 30.2 ± 1.9, *p* = 0.24). Overall diastolic function remained unchanged during the observational period. Only echocardiographic measured E/A-ratio showed a transient decrease after cancer therapy, whereas E/ E’ remained stable after 12 months (Fig. 3 H + I). These findings are supported by CMR derived LAVi and RAVi measurements and even computed PCWP, which did not change during the observational time (Fig. 3J-L).

### Assessment of myocardial tissue characteristics

As can be seen in Fig. 3D, the ratio of myocardial-mass and body-surface-area increased initially after cancer therapy. This was accompanied by a significant rise in T2 times, whereas T1 times increased numerically but not statistically significant (Fig. 3A + C). ECV did not change following cancer therapy (Fig. 3B).

### Change in biomarker

**Fig. 4:**
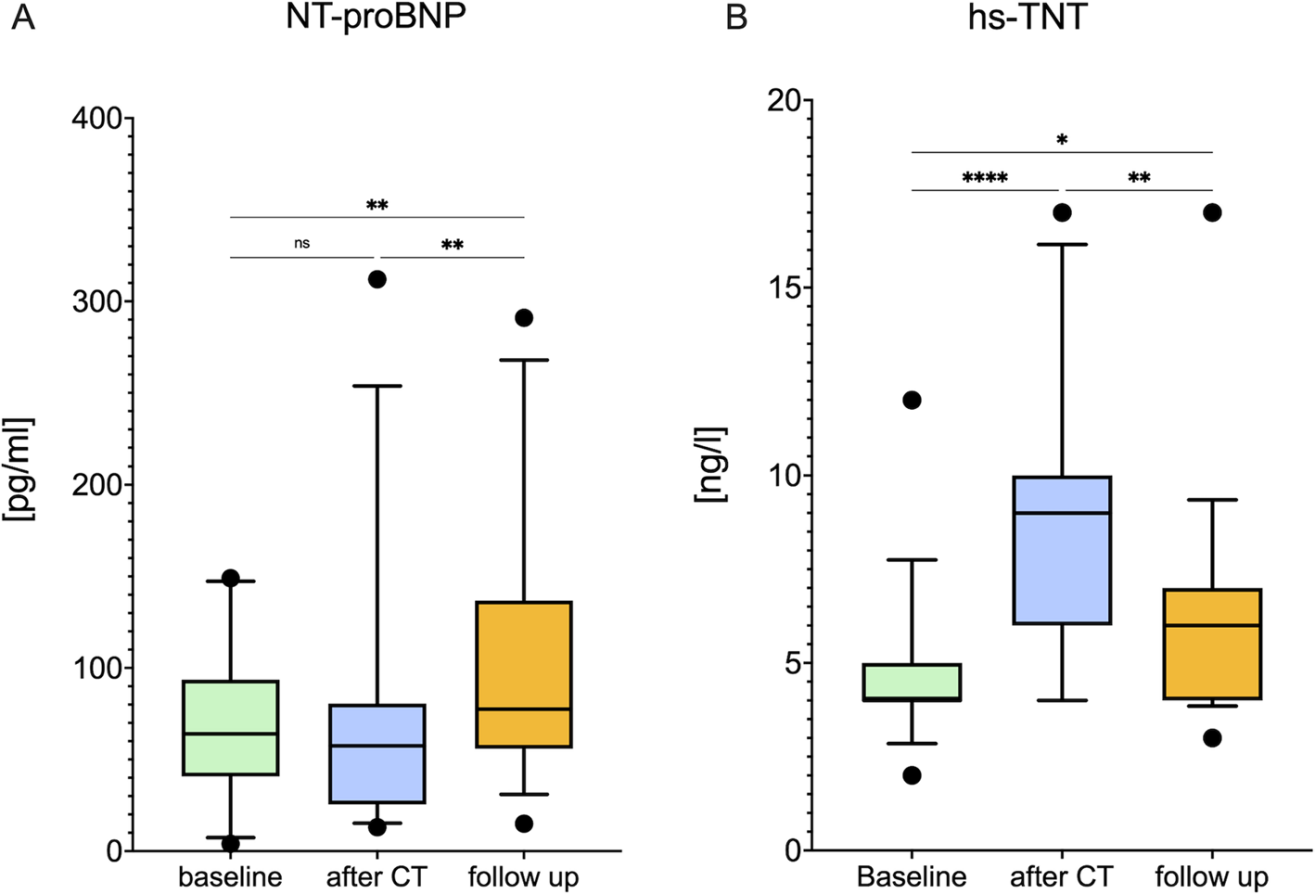
*Anthracycline therapy leads to increased levels of high sensitivity troponin-T and NT-ProBNP.* NTproBNP (**A**) and hs-TNT (**B**) levels were elevated directly and 12 months after cancer therapy. Data are shown as mean with lower and upper quartile, statistics are performed by ANOVA for repeated measurements followed by Tukeys post-hoc analyses respectively by Friedmanns test followed by Dunn’s post-hoc analyses in cases of non-normal distribution, *n* = 59. hs-TnT = high sensitive Troponin T, NT-pro BNP = NT-pro brain natriuretic peptide, *: *p* < 0.05, **: *p* < 0.01, ***: *p* < 0.001, ****: *p* < 0.0001. Figure created with GrahPad Prism 9 for macOS

Baseline levels of hs-TnT (4 [4;5] ng/l) and NT-proBNP (64 [40.8;93.5] pg/ml) were below the 99th percentile of the upper reference limit. NT-proBNP levels increased during the follow up period (57.5 [56;136] pg/ml) and remained elevated (77.5 [56; 137] pg/ml, Fig. 4A). Directly (9 [6;10] ng/l) and 12 months after cancer therapy (6 [4;7] ng/l) median hs-TnT levels were higher than at baseline but still below the 99th percentile of the upper reference limit (Fig. 4B).

### Clinical outcomes and symptoms

After cancer therapy 3 patients (5%) presented with acute chest pain and 22 (37,3%) with new onset of dyspnea during exercise, described as NYHA stage II and 2 (3,4%) with NYHA stage III (Supp. table 1, Suppl. Figure 2). After 12 months only 2 patients described dyspnea, both NYHA stage II (Suppl. Figure 1). 3 patients (5%) developed palpations, no patient reported fatigue or syncopes and 2 (3%) were diagnosed with pericardial effusion. New electrocardiographic changes were observed in two women (3.5%), one with changed heart axis the other one with new diagnosed t-wave inversions.

### Incidence of CTRCD detected by CMR according to the 2022 ESC guidelines

**Fig. 5:**
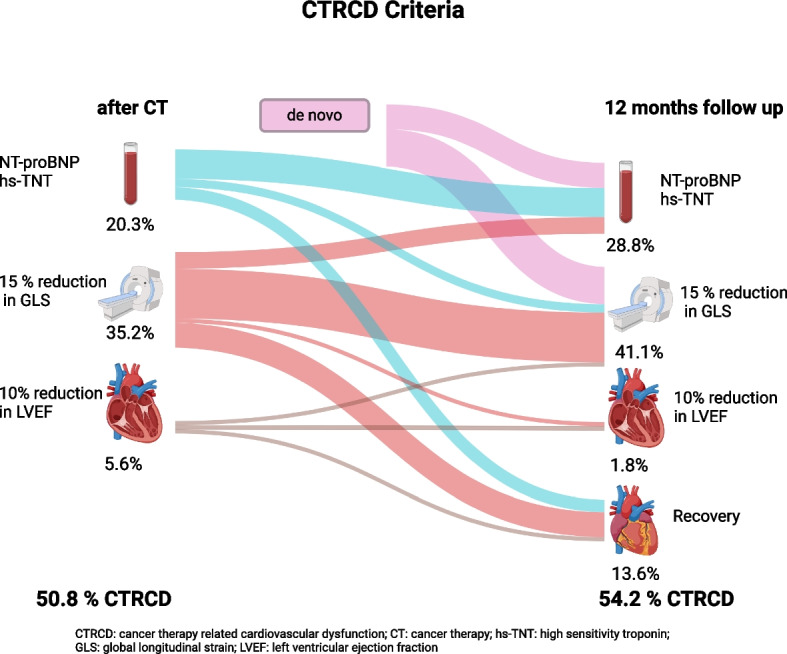
Patients with diagnosed CTRCD directly after CT and after 12 months follow up stratified by underlying CTRCD criteria. Boldness of arrows proportionally represents the number of patients changing between the criteria. Some patients fulfilled more than one criterium for CTRCD. Graphic created with www.biorender.com supported by www.sanceymatic.com. CT: cancer therapy, CTRCD: cancer therapy related cardiovascular dysfunction, GLS = Global longitudinal strain, hs-TnT = high sensitive Troponin T, NT-pro BNP = NT-pro brain natriuretic peptide

According to the 2022 ESC guidelines directly after cancer therapy 30 (50.8%) patients developed CTRCD (at least 15% relative reduction of GLS, new rise in biomarkers or reduction of LVEF < 50%, Suppl. table 2) immediately after cancer therapy, all of them were classified as mild CTRCD, 8 were symptomatic (increased NYHA stage + fulfilled definition of CTRCD (criteria were: EF in 1 patient, impaired GLS in 3 patients and new rise in biomarkers in 4 patients)) and 22 were asymptomatic. Among the asymptomatic patients 19 (32.2%) a relative reduction in GLS of more than 15% and in 12 (20.3%) a new rise in cardiac biomarkers lead to the diagnosis of CTRCD directly after cancer therapy. In total, 3 patients (5.6%) were diagnosed as CTRCD by a LVEF reduction of > 10%, but with an absolute LVEF > 50%. No patient needed to stop cancer treatment or initiate heart failure medication. After 12 months 32 (54.2%) patients fulfilled the criteria for CTRCD, only 2 were symptomatic (with NYHA stage II and impaired GLS in both patients and one of them with additional rise in biomarkers). 23 (41.1%) still had a reduction in GLS > 15%, and in 17 (28.8%) patient´s cardiac biomarkers were newly elevated (Fig. 5). Frequency of patients fulfilling different criteria for CTRCD are displayed in Suppl. Figure 3. Among patients with additional Trastuzumab/ Pertuzumab treatment rates of CTRCD (50%, *p* > 0.99) were similar to the overall cohort.

T2-times after cancer therapy showed only modest accuracy in prediction of CTRCD after 12 months by ROC-analysis (AUC: 0.69, Suppl. Figure 4).

## Discussion

The present study investigated for the first-time low risk patients undergoing anthracycline-based cancer therapy for breast cancer treatment by a structured CMR based follow up with strain analysis and T1- and T2-Mapping for in depth characterization of myocardial damage.

Our main findings were as follows:

1. An initial decline and partly recovery of CMR-measured LVEF was present in every single (investigated) patient undergoing anthracycline-based cancer therapy and was beyond the detection limits of echocardiography.
2. While diastolic function remained preserved, transiently impaired myocardial contractility was coincided by an increase of myocardial mass and T2-values.
3. According to the 2022 ESC guidelines 50.8% of (low risk) patients developed mild CTRCD directly after cancer therapy based on strain analysis by CMR (GLS) or biomarkers, 26.6% of them were classified as symptomatic CTRCD. Even after 12 months 54.2% of patients were diagnosed with mild CTRCD based on strain analysis or biomarkers.

We demonstrate a systematic common subclinical myocardial contractility impairment in every single (investigated) individual undergoing anthracycline-based cancer therapy, although only low risk patients according to the ESC endorsed HFA-ICOS score were included. To the best of our knowledge, no other study investigated this subgroup of patients on an individual basis by multiparametric CMR with a follow up of 12 months. A study by Gulati et al. [14] investigating the effects of candesartan and metoprolol in patients receiving anthracycline-based cancer-therapy included 30 patients treated with placebo but did not analyze T1- or T2-time nor report LVEF changes on an individual basis. A follow up was reported after 24 months only. Recently an additional analysis of this trial using the current criteria to define CTRCT by current criteria has been published, reporting incidences of CTRCD which go in line with our results [15]. Additionally, Lopez-Sendon et al. [16] prospectively investigated patients with different cancer types who received varying therapies demmed high risk for CTRCD and reported high incidences of CTRCD. However, their cohort was more heterogenous, CMR was not used, and cardiovascular risk-factor were frequent among these patients. Nevertheless, comparisons between these different cohorts may help to guide future screening algorithms for CTRCD. Overall, reported data on CTRCD in this particular subgroup remains sparse [17] but childhood cancer survivors treated with anthracyclines showed signs of myocardial damage compared to healthy controls but without longitudinal observation [18].

### Clinical implications

According to the recent definition more than 50% of patients in our cohort fulfilled the criteria of mild CTRCD as detected by CMR although being initially classified as low risk patients. In contrast to that no patient suffered CTRCD when using older definitions of CTRCD (> 10% of LVEF reduction below 50% LVEF) [19]. These results underline the intention of the current guidelines of early and accurate detection of CTRCD, although until now no therapeutic implication is made for mild cases.

With a mean reduction of 6 ± 3% and only a few patients with a decrease of > 10% in LVEF and an absolute LVEF > 50% as measured by CMR one might consider these changes as clinically negligible but since our cohort is at a mean age of 45.5 ± 9.47 years these changes may impact their quality of life, intensify upcoming cardiovascular diseases, or even influence decisions on cancer therapies in the future. In detail, patients who were diagnosed with earlier CTRCD are classified as very high-risk patients for future therapies not only with anthracyclines but also with many other cancer therapeutic agents according to the current guidelines. Additionally, 12 months after cancer therapy four patients were newly diagnosed as borderline LVEF (between 50–54%), which is considered a moderate risk factor for future therapies. Of note, borderline LVEF as a risk factor has only been introduced as an expert consensus (level of evidence C). As a direct therapeutic consequence of these risk estimations ACE-inhibitors, betablockers and statins should be considered in these patients in a case of repeated therapies with different cancer treatments and more importantly interdisciplinary discussion before initiation of cancer treatment is indicated [9, 20–22]. But all these considerations are made mainly due to evidence from older patients who often presented with cardiovascular risk factors. In our opinion these classifications should be further validated for young patients without risk factors in future studies.

### Decreased myocardial function and myocardial tissue characteristics

Reduction in LVEF was mainly driven by ESVi increase without regional differences and accompanied by a transient myocardial edema measured as increased T2 times and elevated myocardial mass, which goes in line with previous investigations after anthracycline therapies [23, 24]. Since the T1-times remained stable after 12 months in our cohort and also earlier studies one might refer the observed myocardial damage as transient decrease in LVEF. Nevertheless, NT-proBNP levels were increased and GLS remained impaired until the end of our study, suggesting a chronification with major clinical impact since CMR derived GLS impairment has been reported as predictor for cardiovascular mortality [25]. In contrast levels of hs-TNT and T2-times, as markers of acute myocardial damage, normalized in the course in our cohort.

Absolute T2-times measurements directly after cancer therapy showed only modest accuracy in predicting CTRCD after 12 months in our cohort, which may rely on the relatively low threshold to diagnose CTRCD and the timepoint of CMR and goes in line with recent reports [26]. Galan-Arriola et al. [23] reported first changes in T2-times already two weeks after first cancer therapy cycle. Although, in this study, heart rate increased to compensate for reduced stroke volume index shortly after cancer therapy cardiac index and resting cardiac power index decreased slightly over time. These changes underline manifest hemodynamical effects induced by cancer therapy in these otherwise healthy women.

Whether myocardial damage could have been limited by early initiation of heart failure therapies such as ACE inhibitors or betablockers in those low risk patients needs further investigation, but since a recent meta-analysis among randomized controlled trials including the whole risk spectrum of patients points towards beneficial effects of these therapies [27], one may speculate that these would also help in low risk patients.

### CMR and echocardiographic imaging

CMR was able to detect a drop in LVEF among all patients whereas echocardiographic measurements were not able to detect these small changes consistently, which goes in line with previous investigations which describe CMR as gold standard for LVEF estimation. Importantly our results reflect clinical practice since echocardiography was performed by the physician in charge at the echocardiography laboratory at the timepoint of the investigations. That is why examiners differed between patients and more importantly between the timepoints, which may have led to interobserver variability and consequently impeded the detection of small changes in LVEF. But nevertheless, this reflects clinical practice in most cases and is therefore crucial to consider when defining cut-off for CTRCD concerning different imaging modalities.

### Limitations

Several limitations must be considered, first of all the relatively small number of patients included in our study does not really allow general conclusions, but at least patients were recruited from two different hospitals and only few data investigating patients with low risk patients after anthracycline treatment in a longitudinal manner exists so far. Second echocardiographic data were not available for all patients and echocardiographic investigations were conducted by different investigators, GLS measurements by echocardiography were only available in 61% of cases, mostly due to medical reasons (bad image quality, pain or tumor). A bigger sample size may have allowed to detect differenced even by echocardiography. But indeed, this reflects clinical practice in most centers. In addition, preexisting but unknown risk factors may have been rarely present in individuals since patients were included to their medical history. Finally, no conclusion can be drawn about a follow up longer than 12 months due to the limited timeframe of our study.

## Conclusion

Anthracyclines induce a in part subclinical and transient decline of myocardial contractility in every single (investigated) breast cancer patient even without pre-existing risk factors for CTRCD, which may lead to an increased risk of future cardiovascular diseases in these patients.

### Supplementary Information

Supplementary Material 1: Supplemental Figure 1. Types of chemotherapy among included patients*. *All patients (*n*=59) received the standard treatment regimen of epirubicin + cyclophosphamide (4 x) and paclitaxel (12 x). Patients with triple-negative receptor status received carboplatin (*n*=11) and those with positive HER2 receptor statusreceived trastzumab/pertuzumab, additionally. n=59. Figure created with GrahPad Prism 9 for macOS.

Supplementary Material 2: Supplemental Figure 2. Changes in NYHA-Stages over time.NYHA stage at baseline, directly after cancer therapy (after CT) and 12 months after cancer therapy (follow up), *n*=59. Figure created with GrahPad Prism 9 for macOS.

Supplementary Material 3: Supplemental Figure 3. Frequency of overlapping CTRCD defining Criteria. Frequency of overlapping CTRCD defining critereia directly after cancer therapy (A) and after 12 months follow up (B). LVEF: left ventricular function, GLS = Global longitudinal strain, Figure created with GrahPad Prism 9 for macOS.

Supplementary Material 4: Supplemental Figure 4. Prediction of CTRCD by T2-times.T2-times directly after cancer therapy showed a modest predictive value for CTRCD 12 months after caner therapy by ROC-Analysis (AUC: 0.69, *p*=0.02).

Supplementary Material 5:  Supplemental Table 1. Clinical presentation immediately after cancer treatment. According to the 2022 ESC guidelines directly after cancer therapy 30 (50.8%) patients developed CTRCD, all of them were classified as mild CTRCD, 8 were symptomatic. CT: cancer therapy; hs-TNT: High-Sensitivity Troponin T; NT-proBNP: N-terminal pro B-type natriuretic peptide. Supplemental Table 2. Definition of cancer therapy-related cardiac dysfunction (CTRCD) as adopted from the 2022 ESC Guidelines on Cardio-Oncology CTRCD: cancer therapy-related cardiac dysfunction, HF: heart failure LVEF: left ventricular ejection fraction.

## Data Availability

No datasets were generated or analysed during the current study.

## Abbreviations

CMR: Cardiovascular magnetic resonance
CTRCD: Cancer therapy-related cardiac dysfunction
EDV(i): End-diastolic volume (index)
ESV(i): End-systolic volume (index)
GCS: Global circumferential strain
GLS: Global longitudinal strain
GRS: Global radial strain
LAV(i): Left atrial volume (index)
LVEF: Left ventricular ejection fraction
PCWP: Pulmonary capillary wedge pressure
