# Supplementary figures and images for: Anthracycline therapy induces an early decline of cardiac contractility in low-risk patients with breast cancer
Source: Cardiooncology. 2024 Jul 16;10:43. doi: 10.1186/s40959-024-00244-y (PMC11251313; doi:10.1186/s40959-024-00244-y)

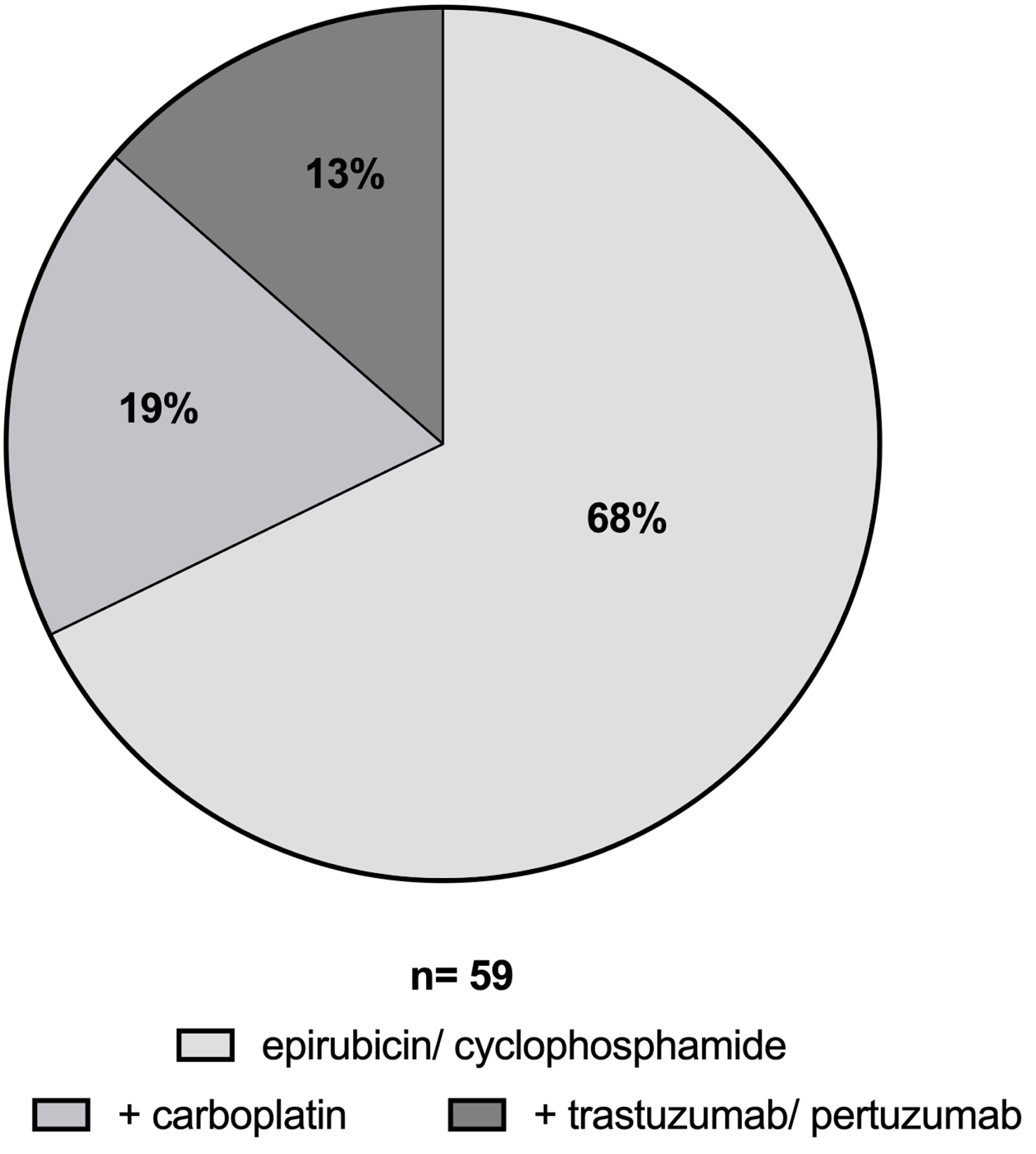

Supplement: Supplementary file 1 — Supplementary Material 1: Supplemental Figure 1. Types of chemotherapy among included patients. All patients (n=59) received the standard treatment regimen of epirubicin + cyclophosphamide (4 x) and paclitaxel (12 x). Patients with triple-negative receptor status received carboplatin (n=11) and those with positive HER2 receptor statusreceived trastzumab/pertuzumab, additionally. n=59. Figure created with GrahPad Prism 9 for macOS. [file 40959_2024_244_MOESM1_ESM.tiff]

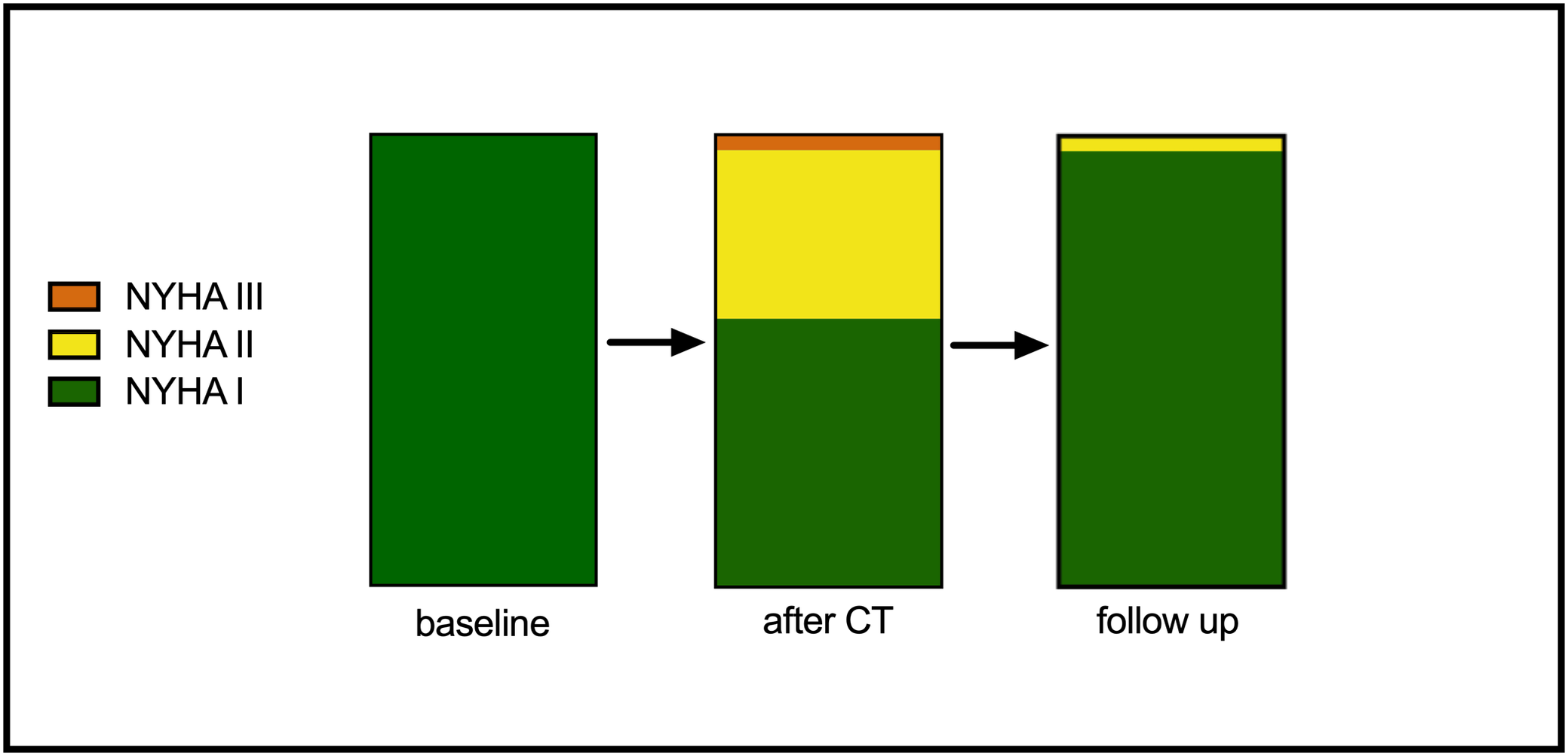

Supplement: Supplementary file 2 — Supplementary Material 2: Supplemental Figure 2. Changes in NYHA-Stages over time.NYHA stage at baseline, directly after cancer therapy (after CT) and 12 months after cancer therapy (follow up), n=59. Figure created with GrahPad Prism 9 for macOS. [file 40959_2024_244_MOESM2_ESM.tiff]

**A****after CT**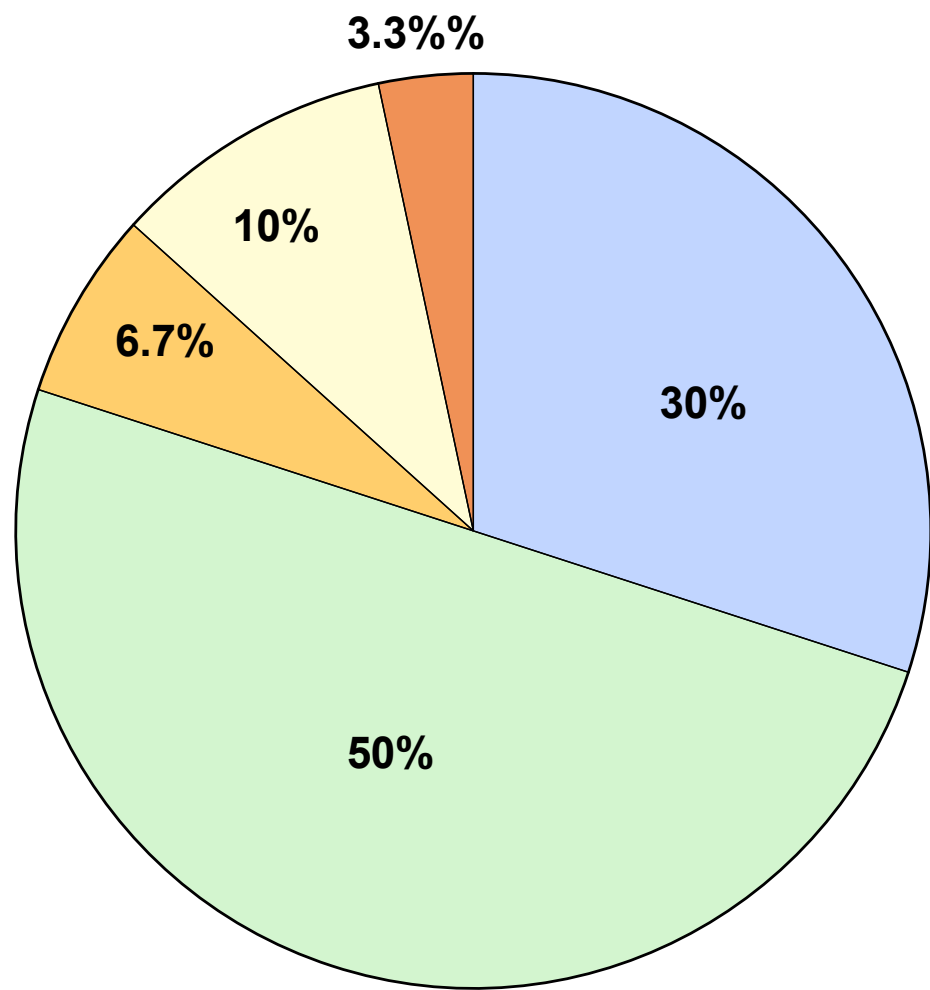**n= 30****B****12 months follow up**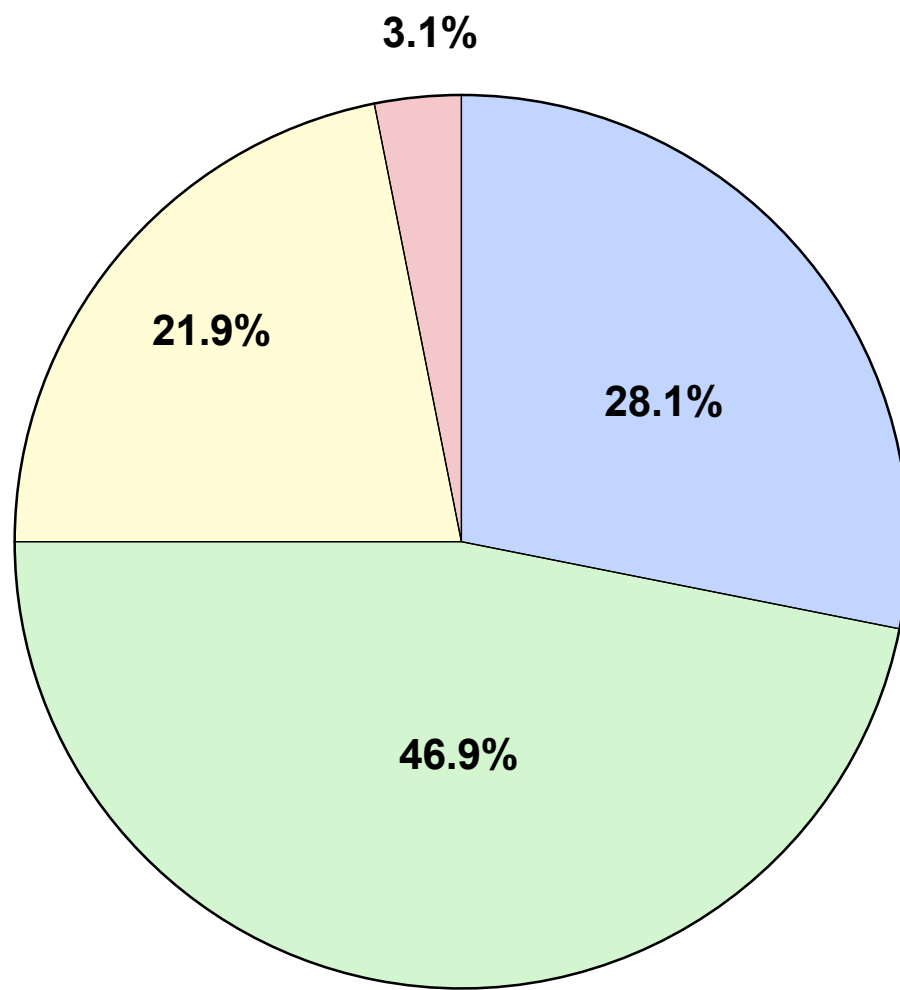**n= 32**

GLS

Biomarker

LVEF

Biomarker + GLS

GLS + LVEF

Biomarker + GLS + LVEF

Supplement: Supplementary file 3 — Supplementary Material 3: Supplemental Figure 3. Frequency of overlapping CTRCD defining Criteria. Frequency of overlapping CTRCD defining critereia directly after cancer therapy (A) and after 12 months follow up (B). LVEF: left ventricular function, GLS = Global longitudinal strain, Figure created with GrahPad Prism 9 for macOS. [file 40959_2024_244_MOESM3_ESM.pdf]

# Prediction of CTRCD by T2-Times

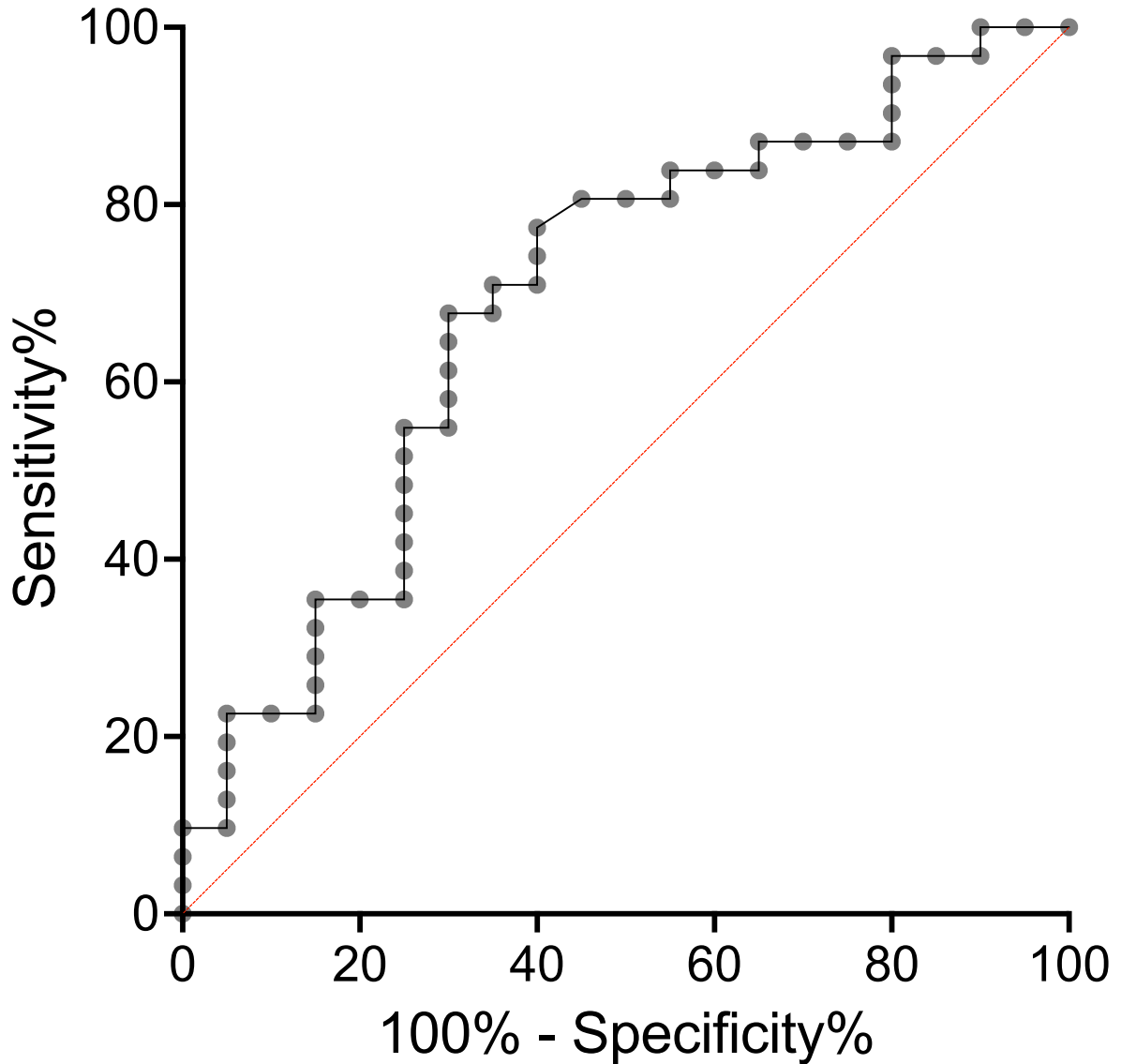

Supplement: Supplementary file 4 — Supplementary Material 4: Supplemental Figure 4. Prediction of CTRCD by T2-times.T2-times directly after cancer therapy showed a modest predictive value for CTRCD 12 months after caner therapy by ROC-Analysis (AUC: 0.69, p=0.02). [file 40959_2024_244_MOESM4_ESM.pdf]
