# Supplementary material for: Anthracycline therapy induces an early decline of cardiac contractility in low-risk patients with breast cancer
Source: Cardiooncology. 2024 Jul 16;10:43. doi: 10.1186/s40959-024-00244-y (PMC11251313; doi:10.1186/s40959-024-00244-y)
Supplement: Supplementary file 5 — Supplementary Material 5: Supplemental Table 1. Clinical presentation immediately after cancer treatment. According to the 2022 ESC guidelines directly after cancer therapy 30 (50.8%) patients developed CTRCD, all of them were classified as mild CTRCD, 8 were symptomatic. CT: cancer therapy; hs-TNT: High-Sensitivity Troponin T; NT-proBNP: N-terminal pro B-type natriuretic peptide. Supplemental Table 2. Definition of cancer therapy-related cardiac dysfunction (CTRCD) as adopted from the 2022 ESC Guidelines on Cardio-Oncology CTRCD: cancer therapy-related cardiac dysfunction, HF: heart failure LVEF: left ventricular ejection fraction. [file 40959_2024_244_MOESM5_ESM.docx]

Supplemental Table 1: Clinical characteristics immediately after cancer treatment

| Clinical characteristics immediately after cancer treatment   \|  \|  \| \| --- \| --- \| \| Variable \| n (%) \| \| Clinical Presentation within one year after CT \|  \| \| Acute Chest Pain \| 3 (5) \| \| New-onset or worsening of dyspnoe at rest or exercise \|  \| \| NYHA II \| 22 (37.9) \| \| NYHA III \| 2 (3.4) \| \| NYHA IV \| 0 (0) \| \| Arrhythmia symptoms; Palpitations, Syncope \| 3 (5) \| \| Fatigue \| 0 (0) \| \| Diagnostic Criteria \|  \| \| Pericardial effusion \| 2 \| \| hs-TNT \| 8.2 ± 3.4 \| \| NT-proBNP level in plasma (pg/mL) \| 68.8 ± 41.2 \| \| Suspicious ECG \| 2 (3.5) \| \|  \|  \| \| Peak Values are represented as mean ± standard deviation,  CT: cancer therapy; hs-TNT: High-Sensitivity Troponin T; NT-proBNP: N-terminal pro B-type natriuretic peptide \| or n (%). \| |
| --- | --- | --- | --- | --- | --- | --- | --- | --- | --- | --- | --- | --- | --- | --- | --- | --- | --- | --- | --- | --- | --- | --- | --- | --- | --- | --- | --- | --- | --- | --- | --- | --- | --- | --- |

Supplemental Table 2: Definition of cancer therapy-related cardiac dysfunction (CTRCD)

| CTRCD as adopted from the 2022 ESC Guidelines on Cardio-Oncology^9^   \|  \|  \| \| --- \| --- \| \| Symptomatic CTRCD \|  \| \| Very severe \| HF treated on intensive care-unit/ cardiogenic shock \| \| Severe \| HF hospitalization \| \| Moderate \| Outpatient intensification of HF therapy/ diuretics \| \| Mild \| Mild HF symptoms without need of intervention \| \| Asymptomatic CTRCD \|  \| \| Severe \| New LVEF reduction to < 40% \| \| Moderate \| New LVEF reduction by ≥ 10 percentage points to an LVEF of 40-49% OR  New LVEF reduction by < 10 percentage points to an LVEF of 40-49% AND either relative decline in GLS by > 15% from baseline OR new rise in cardiac biomarkers \| \| Mild \| LVEF ≥ 50% AND new relative decline in GLS by > 15% from baseline AND/ OR new rise in cardiac biomarkers \| \| CTRCD: cancer therapy-related cardiac dysfunction, HF: heart failure  LVEF: left ventricular ejection fraction \|  \| |
| --- | --- | --- | --- | --- | --- | --- | --- | --- | --- | --- | --- | --- | --- | --- | --- | --- | --- | --- | --- | --- | --- | --- |
